# Computational Fluid Dynamics Study of the Effects of Temperature and Geometry Parameters on a Virtual Impactor

**DOI:** 10.3390/mi13091477

**Published:** 2022-09-05

**Authors:** Ruofei Wang, Heng Zhao, Jiaqi Li, Xingbo Wang

**Affiliations:** Centre for Lidar Remote Sensing Research, School of Mechanical and Precision Instrument Engineering, Xi’an University of Technology, Xi’an 710048, China

**Keywords:** virtual impactor, computational fluid dynamics, air−microfluidic chip, dynamic viscosity, PM

## Abstract

The virtual impactor, as an atmospheric particle classification chip, provides scientific guidance for identifying the characteristics of particle composition. Most of the studies related to virtual impactors focus on their size structure design, and the effect of temperature in relation to the dynamic viscosity on the cut−off diameter is rarely considered. In this paper, a new method that can reduce the cut−off particle size without increasing the pressure drop is proposed. Based on COMSOL numerical simulations, a new ultra−low temperature virtual impactor with a cut−off diameter of 2.5 μm was designed. A theoretical analysis and numerical simulation of the relationship between temperature and the performance of the virtual impactor were carried out based on the relationship between temperature and dynamic viscosity. The effects of inlet flow rate (Q), major flow channel width (S), minor flow channel width (L) and split ratio (r) on the performance of the virtual impactor were analyzed. The collection efficiency curves were plotted based on the separation effect of the new virtual impactor on different particle sizes. It was found that the new ultra−low temperature approach reduced the PM2.5 cut−off diameter by 19% compared to the conventional virtual impactor, slightly better than the effect of passing in sheath gas. Meanwhile, the low temperature weakens Brownian motion of the particles, thus reducing the wall loss. In the future, this approach can be applied to nanoparticle virtual impactors to solve the problem of their large pressure drop.

## 1. Introduction

As a major pollutant in the environment, particulate matter (PM) is composed of solid and liquid particles suspended in the air [1,2]. In recent years, studies on PM2.5 have shown that high concentrations of particulate matter seriously affect the quality of weather, forming a series of bad weather such as haze, which poses a great danger to human health. Epidemiological findings suggest that particulate matter is related to a high risk of cardiopulmonary mortality and morbidity [3,4,5,6,7], that PM exposure poses a significant physical hazard to humans [8,9] and that the severity of the hazard to humans is related to the aerodynamic diameter of PM. Particulate matter can be classified as coarse particles (AD > 2.5 μm), fine particles (AD < 2.5 μm) and ultrafine particles (AD < 100 nm) according to the aerodynamic diameter (AD). Among them, fine particles (PM2.5) may accumulate on the respiratory system, alveoli and other structures [10], resulting in the generation of diseases in the human body. Recently, methods such as compound electric field, thermal precipitation, gravity precipitation, inertial classification and centrifugation [11] have been used for PM separation. Among them, the method of inertial separation is commonly used for PM separation due to its better performance in terms of collection efficiency. Inertial impactors include the conventional, cascade and virtual impactors, which can classify particles into different size ranges according to their inertia. However, conventional impactors have a series of problems such as particle bounce and particle overloading, which in turn affect their collection efficiency performance [12]. First developed in 1966 [13], the virtual impactor has a feature not found in conventional impactors, namely the use of collection probes instead of collection plates to avoid particle bounce. With the emergence of this research, more scholars have begun to investigate virtual impactors. The virtual impactor collection efficiency is influenced by many factors, the most significant of which are the Reynolds number and the Stokes number, with others including the major channel width (S) and the split ratio (r). Marple V.A. et al. determined the properties of the virtual impactor by numerical solution of the Navier–Stokes equations and the particle equations of motion [14]. Chen, T. et al. designed a MEMS−based cascaded virtual impactor for PM2.5 and PM10, and their cascaded virtual impactor collection efficiency curve has a good steepness [15]. Chang et al. proposed a new modified Stokes number based on numerical simulations to predict the cut−off diameter of virtual impactors with different splitting ratios [16]. Wang, Y. et al. designed an aerosol sensor using a 3D printed virtual impactor combined with a SAW sensor [17]. Dong et al. proposed a PM2.5 sensor based on the light scattering method with an integrated virtual impactor [18]. Heo et al. investigated the effect of horizontal inlets on the virtual impactor performance, and their simulations show that horizontal inlets significantly reduced wall loss near the cut−off diameter from about 30% to 3% or less [19]. H. Lee et al. studied the variation of the effect of orifice plates at the inlet on the virtual impactor performance by way of numerical simulations, and their results indicate with the addition of orifice at the inlet, the wall loss can effectively be reduced [20]. Zeeshan Zahir et al. developed an electrodynamic virtual impactor with fine and ultrafine particle sampling capability and evaluated its performance numerically and experimentally. Their results show that by varying the voltage applied to the central electrode, the lower limit cut−off diameter of the major flow channel can be varied. An electrodynamic virtual impactor with variable sampling particle size range was achieved [21]. In summary, most of the studies related to virtual impactors focus on virtual impactor cut−off diameter and wall loss by varying the geometry of the virtual impactor or the inlet flow rate. We found that few studies explore the effect of the ambient temperature inside the virtual impactor on the cut−off diameter. It is shown from the Stokes number equation that the fluid dynamic viscosity is a key factor affecting the cut−off diameter of the virtual impactor, and its correlation with the temperature of the internal environment of the virtual impactor has been little studied so far.

In this paper, we focus on the effect of temperature (T) on virtual impactor cut−off diameter and propose a novel microfluidic chip for ultra−low temperature virtual impactors. This new virtual impactor can reduce its cut−off diameter by introducing ultra−low temperature nitrogen to reduce the internal ambient temperature, which reduces the pressure drop compared to conventional virtual impactors with the same structure and the same cut−off diameter. At the same time, owing to the phenomenon of Brownian motion of fine particles, the virtual impactor has some particles adhering to the wall due to Brownian motion when carrying out particle separation, thus causing wall loss. The intensity of Brownian motion is temperature−dependent, and the lower the temperature, the weaker the Brownian motion. The reduction in the internal temperature of our new virtual impactor makes Brownian motion of particles weaker, which reduces the wall loss of particles to a certain extent and improves the performance of the virtual impactor. Detailed theoretical analysis and numerical simulations are given in Section 2 and Section 3, respectively. We also discuss the effects of inlet flow rate (Q), splitting ratio (r), minor flow channel width (L) and major flow channel width (S) on the performance of the virtual impactor. Finally, we outline a virtual impactor with a cut−off diameter of 2.5 μm and a satisfactory collection efficiency curve steepness.

## 2. Theoretical Analysis and Methods

### 2.1. Theoretical Analysis

**Figure 1:**
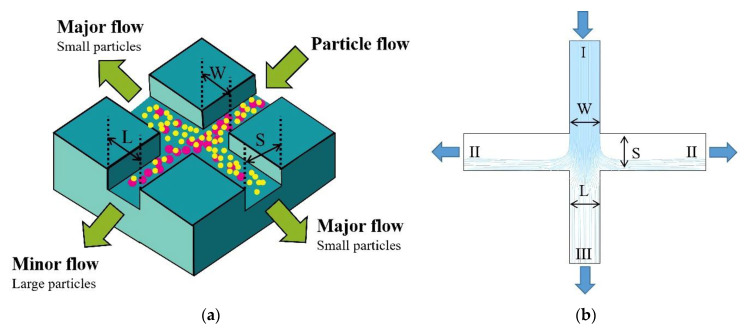
(**a**) 3D model of virtual impactor; (**b**) 2D model of virtual impactor.

When the particles follow the airflow inside the geometric structure, the motion trajectory of the particles is related to their particle size. Theoretically, the fine particles follow the gas streamlines, while coarse particles leave the flow line due to their own inertia [22]. The air−microfluidic chip by this theory separates particles of different sizes, often called the virtual impactor (VI). The distribution of particle trajectories in the VI for particles of different size is shown in Figure 1.

The yellow circles in Figure 1a are fine particles and the pink circles are coarse particles. In Figure 1b, (I) is the airflow and particle inlet, (II) is the major flow outlet, and (III) is the minor flow outlet. Due to the pressure distribution at the outlet, most air flow enters the major flow channel (II), while a small portion of the air flow enters the minor flow channel (III). Particles enter the VI through an accelerated jet (I), and the fine particles follow the fluid movement to the major flow outlet (II). In contrast, the coarse particles maintain linear motion to reach the minor flow outlet (III) due to the influence of their own inertia. The size here refers to the relative cut−off diameter size.

The separation of atmospheric aerosol particles is generally designed based on the Marple theory, and two of the most vital parameters are the Stokes number (Stk) and the Reynolds number (Re) [14].

The Stokes number (a dimensionless number) is defined as the ratio of particle relaxation time to fluid characteristic time, which describes the behavior of suspended particles in a fluid [23]. The efficiency of the virtual impactor (VI) is usually characterized by ${\mathrm{Stk}}_{50}$, which represents the Stokes number when the collection efficiency is equal to 50%. The equation is as follows.

(1) $$\mathrm{Stk}=\frac{\tau U}{W/2}=\frac{\rho_{p}d_{p}^{2}{\mathrm{UC}}_{c}}{9\mu W}=\frac{\rho_{p}d_{p}^{2}{\mathrm{QC}}_{c}}{9{\mu W}^{2}H},$$

where τ is the particle relaxation time, U is the nozzle inlet velocity, $\rho_{p}$ is the particle density, Q is the nozzle inlet flow rate, and $C_{c}$ is the Cunningham correction factor. A previous study pointed out that the pressure at the entrance of the rectangular nozzle (W = H) is the smallest. For a rectangular jet, ${\mathrm{Stk}}_{50}$ is suggested to be 0.59 [18].

Due to the large mean free path of air molecules, its value λ is equal to 0.066 μm in the standard state. In aerosol engineering, the effect of fluid (air) discontinuity is often considered when fine particles settle in the air. In 1910, Cunningham gave the slip correction coefficient $C_{c}$ [22] to correct fluid discontinuity with the equation as follows.

(2) $$C_{c}=\{\begin{array}{c} 1+2.52\frac{\lambda }{d},d>2\lambda \\ 1+3.29\frac{\lambda }{d},d<2\lambda \end{array}.$$

Re (a dimensionless number) is defined as the ratio of the inertia to the viscous force of the fluid, and the equation is as follows.

(3) Re=ρLcUμ=2ρQμ(W+H),

where ρ is the fluid density, Q is the gas flow rate, μ is the aerodynamic viscosity, W is the nozzle inlet width and H is the virtual impactor depth. Since the gas motion in the circulation channel should be laminar, the Re should be in the range of 500 to 3000 [17].

Since the virtual impactor is used as a device for particle size selection, collection efficiency (CE) and wall loss (WL) are used to evaluate its performance. The CE is defined as the ratio of the number of particles at the major flow outlet to the sum of the number of particles at the major flow outlet and minor flow outlet [20]. The WL is defined as the number of particles lost inside the VI, that is, the number of particles adhered to the walls of the VI. It is calculated by dividing the number of particles deposited on the inner wall by the total number of particles at the inlet boundary. The equations for CE and WL are shown below.

(4) $$\mathrm{CE}=\frac{N_{\mathrm{major}}}{N_{\mathrm{major}}+N_{\mathrm{minor}}}\times 100\%,$$

(5) $$\mathrm{WL}=\frac{N_{\mathrm{in}}-N_{\mathrm{major}}-N_{\mathrm{minor}}}{N_{\mathrm{in}}}\times 100\%.$$

### 2.2. Methods

#### Simulation Methods

We used COMSOL Multiphysics software to numerically simulate the laminar flow and particle trajectory tracking and accurately predict the trajectory of the particles in the physical field using finite element analysis [24]. In this paper, we studied particle motion trajectories for different sizes and the collection efficiency of the VI at different conditions. For the numerical simulation, we adopted the 2D model of the VI for laminar flow and particle trajectory tracking [18]. Compared with the 3D model, the number of calculations is greatly reduced, and the simulation results are generally consistent.

Since the air flow is laminar as it moves inside the VI, we chose a laminar flow module (SPF) and a fluid flow particle tracking module (FPT) in the design. In the laminar flow module, COMSOL solves the Navier–Stokes equation and the continuity equation to derive the velocity and pressure distribution of incompressible flow [25].

(6) ρ(u·∇)u=∇·[−pI+K]+F,

(7) ρ∇·u=0,

(8) $$K=\mu \left(\nabla u+{\left(\nabla u\right)}^{T}\right),$$

where ρ(u·∇)u represents the unsteady inertial force, F is the body force, ρ is the fluid density, P is the pressure, I is the unit diagonal matrix, and u is the fluid velocity.

Particle tracking describes the problem in Lagrangian form, where particles are treated as distinct entities rather than as a continuous distribution. In the laminar particle tracking problem, the particles are subjected to the forces of drag, Brownian motion and gravity. The FPT solves the ordinary differential equations with Newton’s law of motion to derive the particle trajectories. The formula is shown below.

(9) $$\frac{d\left(m_{p}v\right)}{\mathrm{dt}}=F_{t},$$

where $m_{p}$ is the particle mass, v is the particle velocity and $F_{t}$ is the combined force on the particle. The combined force includes the forces of drag, Brownian motion and gravity. The equations are as follows.

(10) $$F_{D}=\frac{1}{\tau_{p}}m_{p}\left(u-v\right)=\frac{18\mu }{\rho_{p}d_{p}^{2}}m_{p}\left(u-v\right),$$

(11) $$F_{b}=\xi \sqrt{\frac{12{\pi k}_{B}{\mu \mathrm{Tr}}_{p}}{\Delta t}},$$

where ζ is a Gaussian distributed random variable, $r_{p}$ is the particle radius, $k_{B}$ is the Boltzmann constant, T is the temperature and Δt is the time step. The formula is shown below.

(12) $$F_{g}=m_{p}g\frac{\rho_{p}-\rho }{\rho_{p}},$$

where g is the gravitational gas pedal.

In the SPF boundary condition settings, the inlet condition was set to flow, while the outlet condition was set to pressure, and the wall surface was set to no slip.

In the FPT boundary condition settings, the inlet boundary condition was first set to uniform distribution, and the particle initial velocity was derived from SPF. Both of the wall surface and the outlet boundary should be frozen. Then, the drag, gravity and Brownian motion forces were added. In summary, the above boundary conditions were set as shown in Table 1.

**Figure 2:**
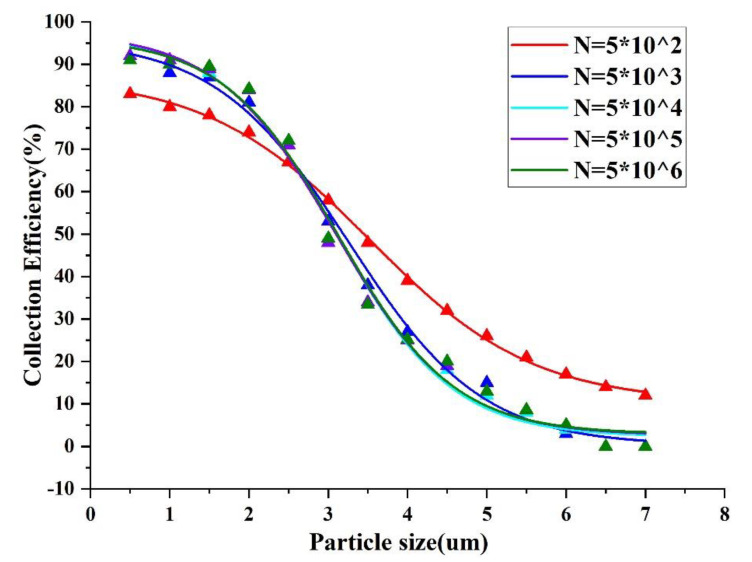
Grid irrelevance verification.

After the design of the key parameters was completed, we performed the grid−independent verification, and N in the figure is the number of grid cells, as shown in Figure 2. As can be seen from Figure 2, the collection efficiency curve tends to be consistent as the number of grids increases. This indicates that the results of the numerical model are reliable with a sufficient number of grids and are not affected by the number of grids. At the same time, considering that the increase in the number of grids increases the operation time, we finally chose the number of grids N equal to $5\times 10^{5}$.

**Figure 3:**
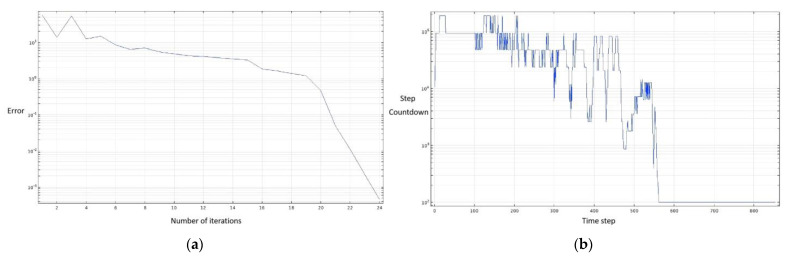
(**a**) Steady−state solver; (**b**) Transient solver.

During the numerical simulation, we have judged its convergence as shown in Figure 3. From Figure 3, it can be seen that the steady−state solver error is less than $10^{-3}$, and the transient solver convergence plot shows a decreasing trend, so the numerical model converges.

In order to obtain a VI with a cut−off diameter of 2.5 μm, the $d_{50}$ was first set to 2.5 μm, and the Cunningham correction factor was calculated to be 1.0665. For the subsequent adoption of 3D printing for VI, the nozzle entrance width (W) was temporarily set to 1 mm. The nozzle inlet was set as a rectangle (W = H) as suggested by previous research, while taking the suggested ${\mathrm{Stk}}_{50}$ of 0.59 [18]. At this point, the nozzle inlet flow rate Q was calculated according to the Stokes number equation as 0.86 L/min, which means that the inlet flow rate U was 14.4 m/s. Substituting U into the Reynolds number equation yields Re = 959, which is in the laminar flow range. The final values of the key virtual impactor parameters are shown in Table 2.

In the numerical simulation, the SPF and the FPT were calculated separately. The flow field was first calculated, and the particle trajectory was calculated after the flow field reached a steady state. This calculation method has the advantage that when the particle size needs to be altered and re−simulated, only the FPT needs to be calculated, and the flow field does not need to be recalculated, saving computation time.

## 3. Results and Discussion

### 3.1. Flow and Pressure Analysis

**Figure 4:**
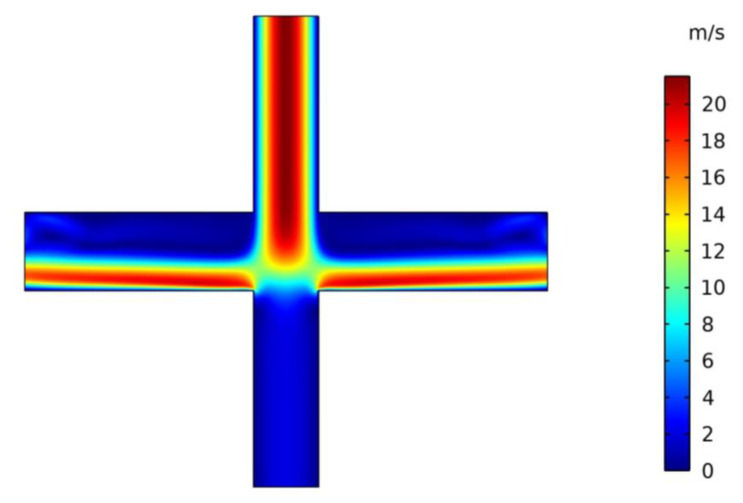
Velocity distribution in VI.

The VI velocity distribution at an inlet flow rate Q of 0.86 L/min is shown in Figure 4. It can be seen that the VI velocity is symmetrically distributed, and for the major flow channel the velocity is larger than that for the minor flow channel.

**Figure 5:**
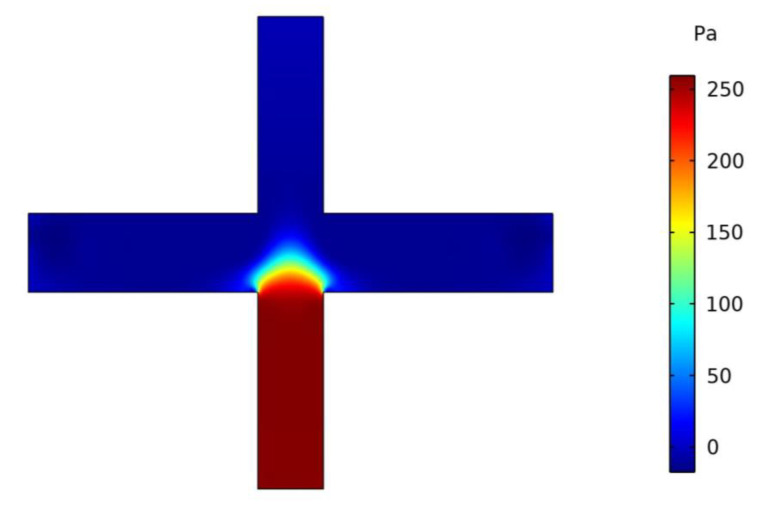
Pressure distribution in VI.

Figure 5 shows the pressure distribution inside the VI. As can be seen in Figure 5, the minor flow channel pressure is much greater than that of the major flow channel, and there is a good pressure difference between the channels. The pressure difference is used to control the flow at the minor flow channel outlet, so that the minor outlet flow is less than 10%, which makes the VI wall loss relatively small [26]. Meanwhile, the low pressure at the inlet facilitates the reduction in energy consumption.

**Figure 6:**
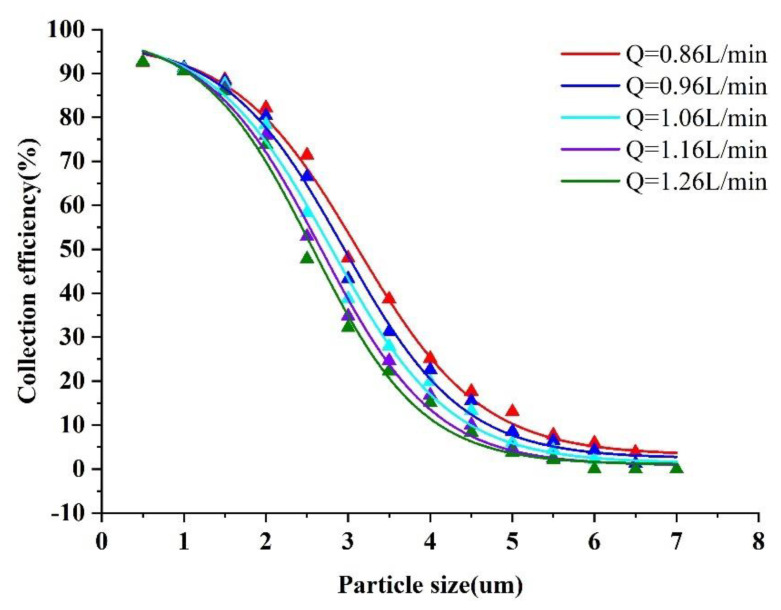
Collection efficiency curves at different Q values.

Figure 6 shows the collection efficiency curve at various inlet flows, and it is clear that the cut−off diameter decreases with the increase in the inlet flow rate Q. At the same time, for the prototype configuration, the Stokes numbers corresponding to different flows are all around 0.77. Considering that the flow rate increase causes an increase in pressure inside the virtual impactor, the final inlet flow Q of 0.86 L/min was selected to obtain a low−pressure virtual impactor.

### 3.2. Effect of the Minor Flow Channel Width (L) and the Major Flow Channel Width (S) on Col−Lection Efficiency

**Figure 7:**
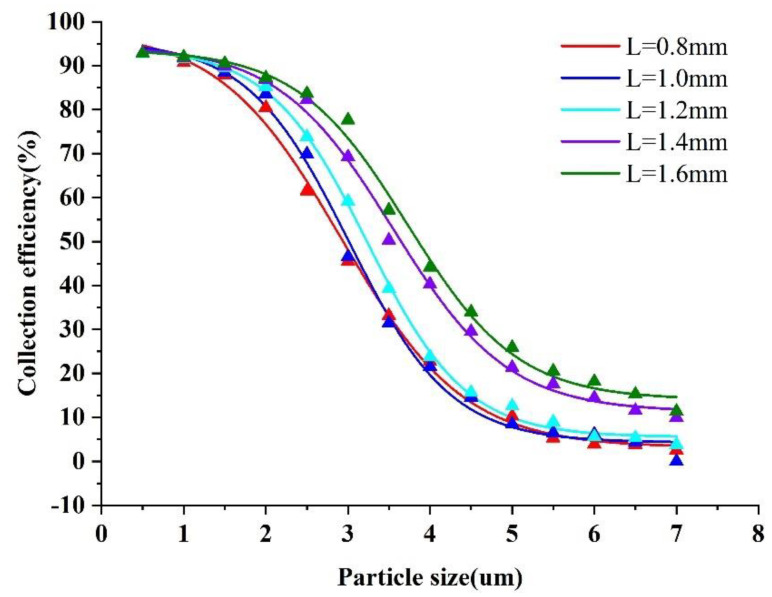
Collection efficiency curves at different L values.

**Figure 8:**
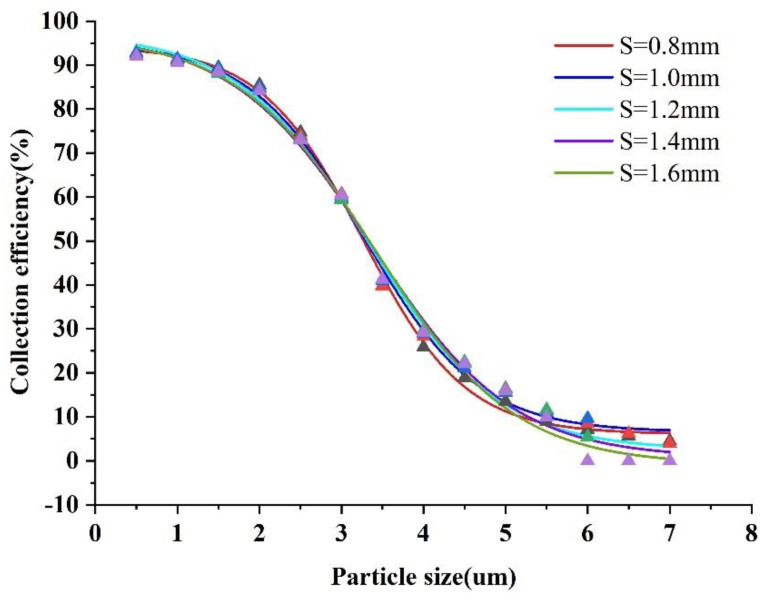
Collection efficiency curves at different S values.

The collection efficiency curves were obtained by varying the width of the minor flow channel (L) and the major flow channel (S). The trajectories with different sizes of particles were simulated and are shown in Figure 7 and Figure 8.

As can be seen in Figure 7, different minor channel widths affect the collection efficiency significantly. The corresponding cut−off diameter increases with the increase in the minor flow channel width. In terms of results, the steepness of the collection efficiency curve that is best for L is equal to 1.0 mm, and the final value of L was chosen as 1.0 mm.

As can be seen in Figure 8, the major flow channel width (S) has little effect on the virtual impactor collection efficiency. In conclusion, the collection efficiency curves under different S values basically overlap, while the steepness is not much different. The collection efficiency curve has a better steepness when S is 1.4 mm, and the final S value was chosen to be 1.4 mm. This value is in line with the empirical range; namely, S is equal to 1.2–1.8 W.

**Figure 9:**
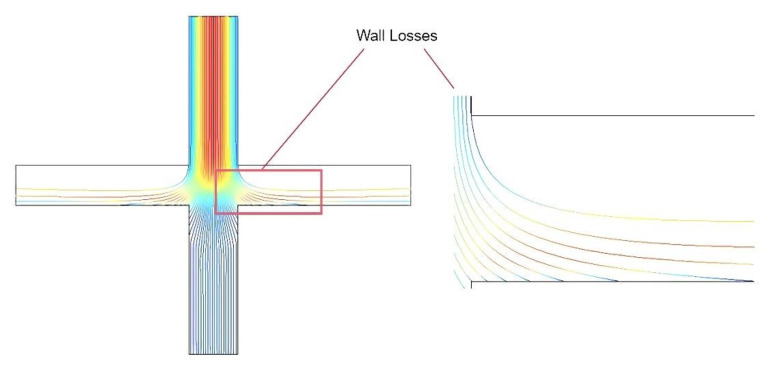
Wall loss on the major flow channel.

At the same time, we found that many particles with size values near the cut−off diameter as shown in Figure 9 adhered directly to the wall of the major flow channel, causing wall loss inside the VI. This is possibly because these particles do not have a sufficiently high inertia to escape the streamlines to enter the minor flow channel, while the particles cannot follow the laminar flow directly to the major flow outlet due to the small space in the major flow. Thus, we designed the major flow channel as a trapezoidal structure, which allows for more space in the major flow path and reduces wall loss.

**Figure 10:**
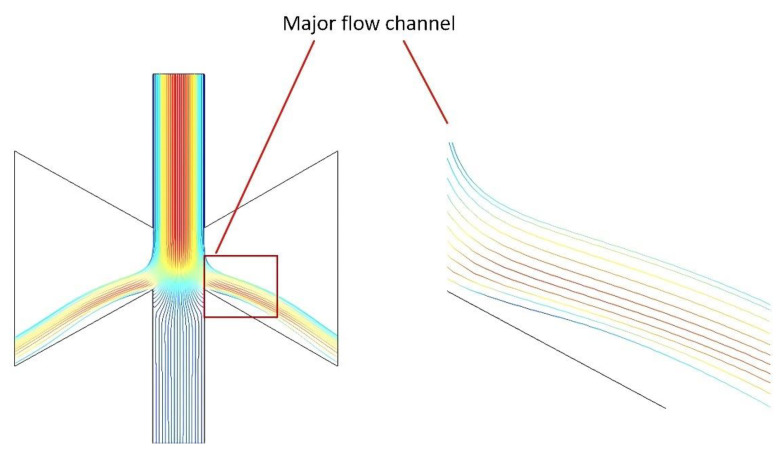
Trapezoidal major flow channel particle motion trajectory.

The colors in Figure 10 represent the particle velocity, with red representing the maximum velocity and blue representing the minimum velocity. It can be seen from Figure 10 that the trapezoidal major flow channel structure allows enough space for the particles to follow the laminar flow to the outlet, which avoids particles sticking to the major flow channel walls due to insufficient inertia. Compared with the rectangular major flow channel, the number of particles reaching the major flow outlet is increased, which reduces wall loss and improves collection efficiency.

### 3.3. Effect of the Split Ratio (r) on Collection Efficiency

**Figure 11:**
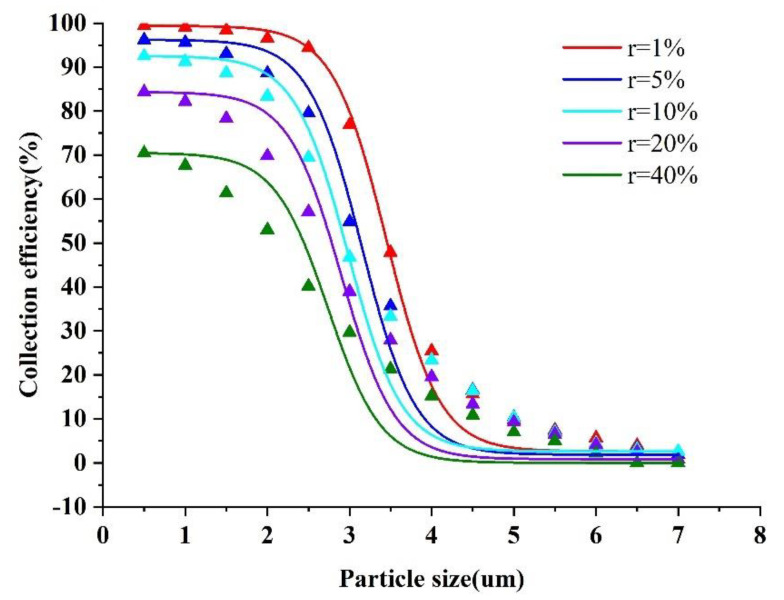
Collection efficiency curves at different split ratios (r).

The splitting ratio r is defined by the ratio of the minor flow outlet flow to the nozzle inlet flow. The collection efficiency curves obtained by varying the splitting ratio (r) while simulating the trajectory of particles of different sizes are shown in Figure 11.

It is obvious from Figure 11 that the splitting ratio has a great effect on the collection efficiency, and the collection efficiency curves decrease in steepness as r increases. When r is equal to 40%, the collection efficiency of particles has a maximum of 70%, indicating that the wall loss of the VI also increases with the increase in r, which is unfavorable to the performance of the VI. Accordingly, r should not be too large. From the results in Figure 11, the collection efficiency curve has a better steepness at r which is equal to 10%. When r is further reduced, the collection efficiency curves change slightly in steepness, but the cut−off diameter gradually increases, which is unfavorable to design the virtual impactor with a low cutting point. Therefore, the final choice of r value is equal to 10%.

### 3.4. Effect of the Temperature (T) on Collection Efficiency

**Figure 12:**
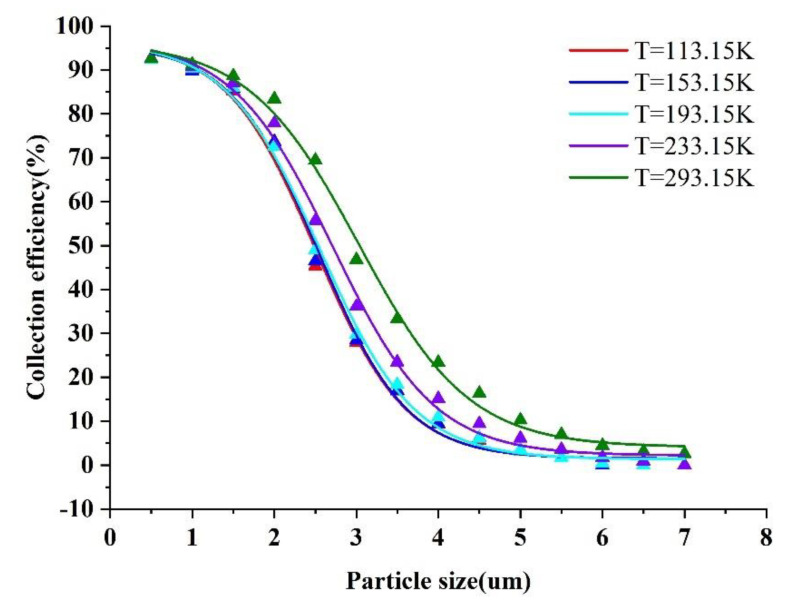
Collection efficiency curves at different temperature (T).

According to the Stokes number equation, it can be seen that the fluid dynamic viscosity is one of the influencing factors, while the aerodynamic viscosity is related to the temperature and almost independent of the pressure. The aerodynamic viscosity can be calculated using the Sutherland equation when the temperature is less than 2000 K [27].

(13) $$\frac{\mu }{\mu_{0}}={\left(\frac{T}{288.15}\right)}^{1.5}\frac{288.15+B}{T+B},$$

where $\mu_{0}$ is equal to $1.7894\times 10^{-5}$ (viscosity at 15 degrees Celsius), and B is a constant related to the type of gas; for air, B = 110.4 K. From Equation (13), when the temperature T decreases, the fluid dynamic viscosity μ decreases. Hence, after we designed the virtual impactor based on a determined number of ${\mathrm{Stk}}_{50}$, the fractional equation on the right side of Equation (1) was constant. When the temperature (T) decreases, the dynamic viscosity (μ) of the fluid decreases, causing the cut−off diameter ($d_{50}$) to decrease. The collection efficiency curves obtained by varying the temperature (T) while simulating the trajectory of particles of different sizes are shown in Figure 12.

It can be seen from Figure 12 that the cut−off diameter decreases with decreasing temperature, and the effect is more obvious when the particle size is relatively large. There is no substantial difference in the effect of the cut−off diameter when the temperature is between 113.15 and 193.15 K, and the CE curve almost overlaps. The steepness of the CE curve improves as the temperature decreases, indicating that low temperature has a significant effect on the performance improvement of the VI. In summary, the final temperature T was chosen to be 193.15 K. When the inlet flow rate Q is 0.86 L/min and the temperature is reduced from 293.15 to 193.15 K, the cut−off diameter is reduced from 3.1 to 2.5 μm, a reduction of about 19% at this time. One study points out that the introduction of sheath gas to a virtual impactor with a cut−off diameter of 2.5 μm can reduce its cut−off diameter to 2.1 μm, a reduction of about 16% [28]. The effect of temperature on the virtual impactor was found to be non−negligible in comparison with the effect brought about by the temperature change mentioned above.

As the particles are affected by Brownian motion, the virtual impactor has some particles adhering to the wall due to Brownian motion during particle separation, thus causing wall loss. The temperature of Brownian motion is related to the degree of intensity, and the higher the temperature, the more intense Brownian motion. The reduction in the internal temperature of our new ultra−low temperature virtual impactor weakens Brownian motion of particles, reducing the probability of particles adhering to the wall due to Brownian motion and improving the performance.

Another advantage of using temperature to reduce the cut−off diameter is that the pressure distribution inside the virtual impactor does not change when the cut−off diameter decreases due to the decrease in temperature. Compared with other virtual impactors, our new ultra−low temperature virtual impactor has a relatively small pressure at the same cut−off diameter, achieving the purpose of reducing the pressure drop with the same cut−off diameter.

For the prototype configuration, the temperature is reduced by 100 K, and the cut−off diameter is reduced by 0.5 μm, which is 500 nm. The conventional virtual impactor has a large pressure drop when classifying particles based on size, especially for ultra−fine particles with diameters less than 100 nm. Therefore, most virtual impactors for classification of particles larger than 100 nm also have cut−off diameters determined by the geometry of their flow channels. To address this issue, we can reduce the cut−off diameter of the virtual impactor with a cut−off diameter greater than 100 nm to less than 100 nm by means of ultra−low temperature, so that we can obtain a smaller cut−off diameter while the pressure drop on the virtual impactor does not increase.

In the actual design, we can use ultra−low temperature nitrogen to obtain a low temperature environment, which is achieved by passing in aerosol particles along with nitrogen gas. This design can ensure that the virtual impactor is always kept at a low temperature during the entire process of particle separation, which helps to guarantee the stability of the virtual impactor performance.

### 3.5. Result of the Simulation

**Figure 13:**
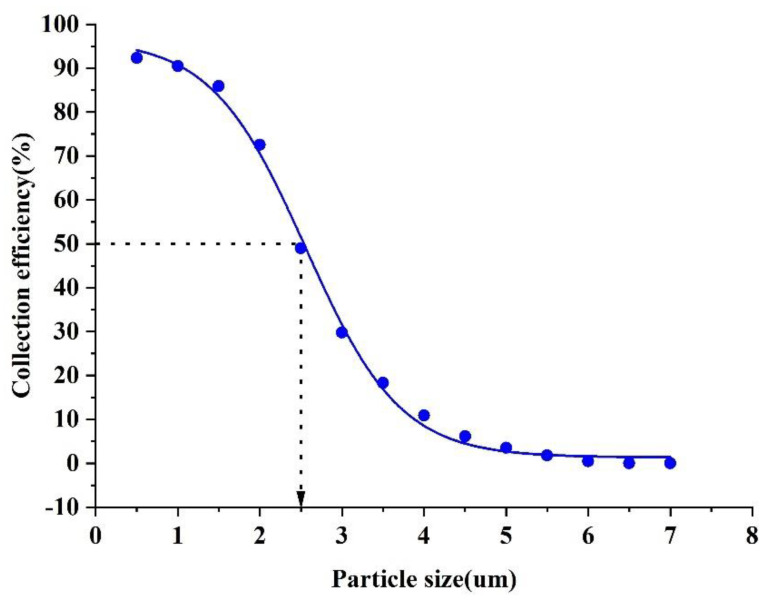
New ultra−low temperature virtual impactor collection efficiency curve.

An ultra−low temperature virtual impactor model was constructed. A new method to reduce the cut−off diameter is proposed. Different sizes of aerosol particles were introduced from the inlet, and the collection efficiency curves of the virtual impactor are shown in Figure 13. The collection efficiency curve shows a cut−off diameter of 2.5 μm and a good steepness of the curve, indicating that the new ultra−low virtual impactor has good performance in separating aerosol particles. Moreover, compared to other methods, the effect of low temperature is slightly better than that of the sheath gas and the orifice. The results are shown in Table 3.

## 4. Conclusions

In this paper, we propose a new method to reduce the cut−off diameter, and we explored the effects of the main structural parameters of the virtual impactor on the performance, such as minor flow channel width (L), major flow channel width (S), inlet flow rate (Q), split ratio (r) and temperature (T) through numerical simulations. We innovatively introduced temperature into the design consideration of the virtual impactor based on the relationship between temperature and dynamic fluid viscosity, and the results of numerical simulations show that temperature has a significant effect on the performance of the virtual impactor. The virtual impactor with a cut−off diameter of 3.1 μm reduces the cut particle size by 19% when the temperature is lowered from 293.15 to 193.15 K, which is slightly better than the effect of the same prototype configuration with the sheath gas inlet. Since most of the nanoparticle virtual impactors have the problem of a large pressure drop, the ultra−low temperature method can be applied to nanoparticle virtual impactors in future work, which provides a new direction for the subsequent research of nanoparticle virtual impactors. We finally obtained a cut−off diameter of about 2.5 μm for the virtual impactor and a collection efficiency curve with good steepness. In future work, we will optimize the performance of the VI by introducing a sheath gas consisting of ultra−low temperature nitrogen. This design mode combines the advantages of sheath gas and a low temperature environment, allowing the performance of the VI to be optimized even further. In summary, there is a sufficient theoretical basis and feasibility to change the cut−off diameter with temperature (T).

## Figures and Tables

**Table 1:** Boundary condition settings.

Boundary	SPF	FPT
Inlet	Flow	Uniform distribution
Outlet	Pressure	Frozen
Wall surface	No slip	Frozen
Compressibility	Incompressible flow	-
Particle initial velocity	-	SPF
Drag force	-	Stokes’ Law

**Table 2:** Variables of the new low temperature virtual impactor design.

Variable	Value	Unit
W	1	mm
H	1	mm
L	1	mm
S	1.4	mm
r	10%	-
T	193.15	K
Q	0.86	L/min
λ	0.066	μm
$\rho_{p}$	1000	$\mathrm{Kg}/m^{3}$

**Table 3:** Comparison with other methods of decreasing cut−off diameter.

Property	Our Method	Handol Lee et al. [20]	Zeeshan Zahir et al. [28]
Cut−off diameter	2.5 μm	1, 2.5 and 10 μm	2.5, 5 and 10 μm
Method of reducing cut−off diameter	Ultra−low temperature	Orifice	Sheath gas
Reduction effect	19%	16.7%	16%
Curve steepness	Satisfactory	Satisfactory	Satisfactory

## Data Availability

Publicly available datasets were analyzed in this study. These data can be found here: https://pan.baidu.com/s/1-sKnDagfjb8jCWKjpFvLXw, accessed on 5 September 2022 access code: aabb.
